# Fluoroscopy-Guided Splanchnic Nerve Block for Cancer-Associated Pain

**DOI:** 10.7759/cureus.30944

**Published:** 2022-10-31

**Authors:** Amreesh Paul, Anjali Borkar

**Affiliations:** 1 Anaesthesiology, Jawaharlal Nehru Medical College, Datta Meghe Institute of Medical Sciences, Wardha, IND

**Keywords:** alcohol neurolysis, radiofrequency radiation, refractory cancer pain, splanchnic nerves, splanchnic nerve ablation

## Abstract

Pain associated with abdominal malignancies or metastasis can be very severe and can be intractable and resistant to conventional pharmacologic therapies. Typically, narcotics and non-narcotics are used in combination to alleviate the cancer pain, but these are often unsuccessful.

Neurolysis and radio-frequency ablation of the celiac plexus and splanchnic nerves is being used with great success for management of the pain associated with abdominal malignancies with added advantages of improving quality of life, pain relief and decreased narcotic consumption. The tumor or associated lymphadenopathy may result in distortion of the celiac plexus anatomy, thus making it hard to reach the celiac plexus. In such cases, splanchnic nerve block can be employed with relative ease as compared to celiac plexus block.

Given the nature of the debilitating pain associated with these conditions and inadequate pain relief with narcotics, these blocks are a boon in disguise to such patients with altered anatomy. Post administration of the splanchnic block, the functioning and quality of life of patients with abdominal malignancies improve. Hence, these blocks can be used to decrease the morbidity associated with abdominal malignancies.

## Introduction and background

Being diagnosed with cancer is one of the most horrendous things that an individual can undergo. Surviving cancer is just the first step that a patient takes in this journey. One of the major struggles that many patients experience is the pain associated with cancer, and this pain is debilitating in nature, increasing the morbidity. The pain ebbs and flows, often varying widely in intensity, and is accompanied often by a significant acute component as the lesions may progress. One of the most common symptoms that is associated with cancer is pain. The international association for the study of pain defines pain as “an unpleasant, sensory, multidimensional and emotional experience associated with actual or potential tissue damage, or is described in relation to such damage [1].” Pain related to cancer is different from pain experienced by patients without cancer. One-fourth of patients newly diagnosed with cancer, one-third of patients undergoing treatment, and three-fourths of patient with advanced disease experience pain [2-4]. Management of cancer pain can be very challenging and results in a considerable amount of morbidity. The physicians should be adept in accessing and management of cancer pain as it of utmost importance in relieving pain and improving quality of life [5]. Pharmacological and interventional therapies can improve functioning and the quality of life of these patients [6]. The WHO has developed an algorithm that has been widely accepted and is in use [7]. It suggests starting with a non-steroidal anti-inflammatory drug and escalating it to a weak opioid and then to a strong opioid if pain relief is not adequate. 

Pain associated with abdominal malignancies or metastasis can be very severe and can be intractable to conventional pharmacologic therapies [8]. Patients may also experience very unpleasant side effects [9]. Typically, narcotics and non-narcotics are used in combination to alleviate the cancer pain, but these are often unsuccessful. The failure to obtain satisfactory pain relief with pharmacological therapies should be managed with interventional therapies [10]. Neurolysis and radio-frequency ablation of the celiac plexus and splanchnic nerves are being used with great success with added advantages of improving quality of life, pain relief, and decreased narcotic consumption [11,12].

The celiac plexus, the largest visceral plexus, lies deep in the retro-peritoneum and is located over the anterolateral surface of the aorta and around the origin of the truncus coeliacus. It relays nociceptive stimuli from the upper abdominal viscera [13]. In 1914, the technique of blocking the celiac plexus and the splanchnic nerve using percutaneous injection was introduced by Max Kappin. Neurolysis in these two blocks was first described by Robert R. Jones in 1957. The role of neurolysis in pain management from gastrointestinal malignancies was first described by Bridenbaugh [14]. Neurolysis of the celiac plexus is used for abdominal pain relief in abdominal malignancies. However, these blocks are sometimes difficult to perform or are rendered ineffective by the altered anatomy of the celiac plexus due to the tumor itself or associated lymphadenopathy. Hence, neurolysis and/or radio-frequency ablation of the splanchnic nerves can provide a much better pain relief in such patients [15]. 

## Review

Anatomy

The abdominal viscera receives its sympathetic innervation from the anterolateral horn of the spinal cord. The fibres that run from the spinal cord form synapses at the celiac plexus [16]. The greater, lesser, and least splanchnic nerves along with the pre-ganglionic fibres from T5-T12 and ventral roots provide the principal sympathetic (pre-ganglionic) contribution to the celiac plexus. The T5-T9 spinal roots give rise to the greater splanchnic nerve, which travels along the thoracic paravertebral border, enters the crus of diaphragm, and goes into the abdominal cavity, ultimately ending on the celiac ganglion on the same side. The lesser splanchnic nerve travels along the greater splanchnic nerve and it originates from T10-T11 roots. The least splanchnic nerves that pass through the diaphragm to terminate at the celiac ganglion arises from T11-T12 spinal roots [17].

**Figure 1:**
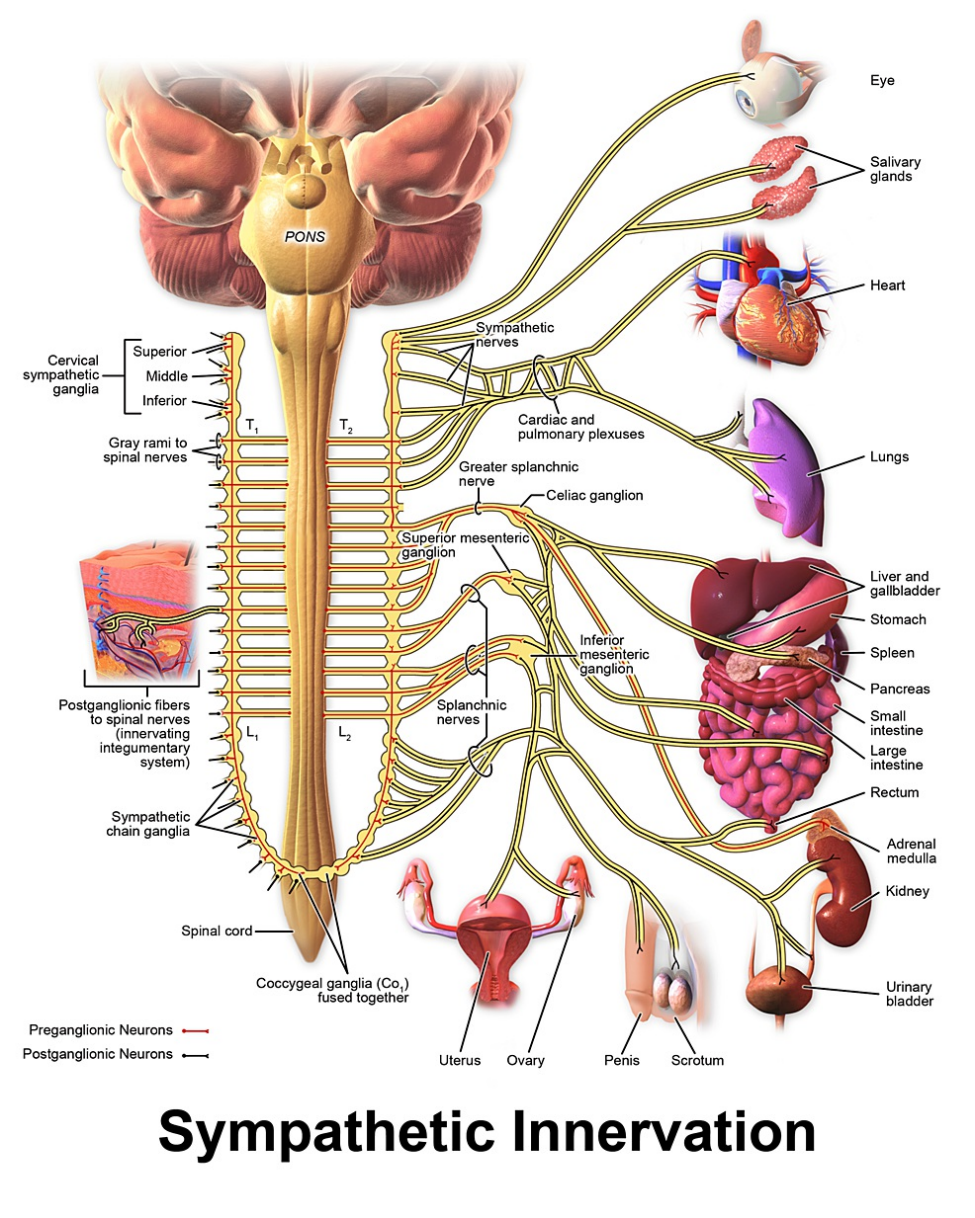
Splanchnic Nerves and Sympathetic Innervation Source: The figure is taken from Biologydictionary.net [21].

All the three splanchnic nerves are pre-ganglionic and form synapses at the celiac ganglion, located anterolateral or anterior to the aorta. There are one to five ganglia in the celiac plexus with a diameter from 0.5 to 4.5 cm [18]. The post-ganglionic fibers form a perivascular plexus along with blood vessels. These fibers are responsible for supplying the abdominal viscera including organs such as the oesophagus, stomach, small intestine, ascending and proximal transverse colon, pancreas, spleen, adrenal glands, and liver (Figure 1). The blockade of all these three nerves is termed as splanchnic nerve block [19,20].

The splanchnic nerves are responsible for the transmission of a major portion of the nociceptive information from the abdominal viscera. They are contained in a compartment that is made up of the pleura and the vertebral body on the lateral side, the attachment of the pleura to the vertebra dorsally, and the posterior mediastinum on the ventral side. The crura of the diaphragm is found caudally to this compartment. The total volume of this compartment is approximately 10 ml on both sides [22].

Coagulation profile of the patient and a fully informed written consent are obtained prior to the procedure. Neurolysis or radio-frequency ablation are carried out only if the patient receives good and temporary pain relief following diagnostic blocks. The patient should be briefed on the potential intra-procedural and post-procedural complications. As the patient might need mild sedation for the procedure, the patients should be Nil per Os as per standard ASA guidelines. A patent intravenous access should be established as there are high chances of hypotension following a successful sympathetic block [23].

Procedure

The patient is usually positioned prone with a pillow under the abdomen and standard monitors are attached. The technique usually is done with the use of a 22G 15 cm Chiba needle or a 22G 9 cm Quincke needle. Blunt-tipped curved needles are preferred as they hug the vertebral body and stay in a medial position. For radio-frequency ablation, the needles should be as long as the needles used for the diagnostic procedures, and must have a 10-15 mm active tip.

After aseptic skin preparation and draping, the T12 vertebral body is identified with the help of the fluoroscope and is squared off. The T11 vertebral body is identified if the diaphragm is found to be overlying the T12 vertebral body. The fluoroscope is angled at a 45-degree angle to the ipsilateral side [24]. After skin and subcutaneous infiltration of local anesthetic, the needle is introduced through the skin. The point of introduction is at the junction of the rib and vertebra just above the lateral border of the vertebral body. In case of bony contact during needle placement, the entry point is placed slightly caudal to the narrowest portion of the vertebral body.

**Figure 2:**
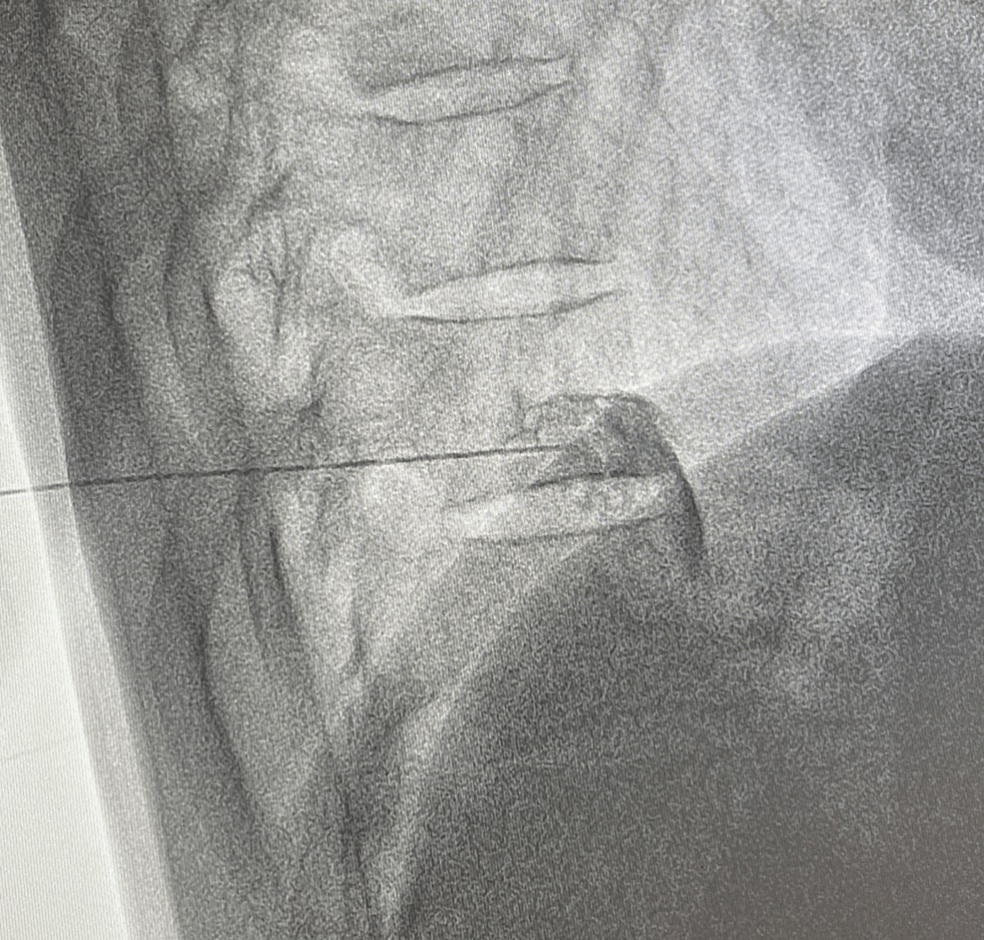
Needle Placement for Splanchnic Nerve Block (Lateral View)

**Figure 3:**
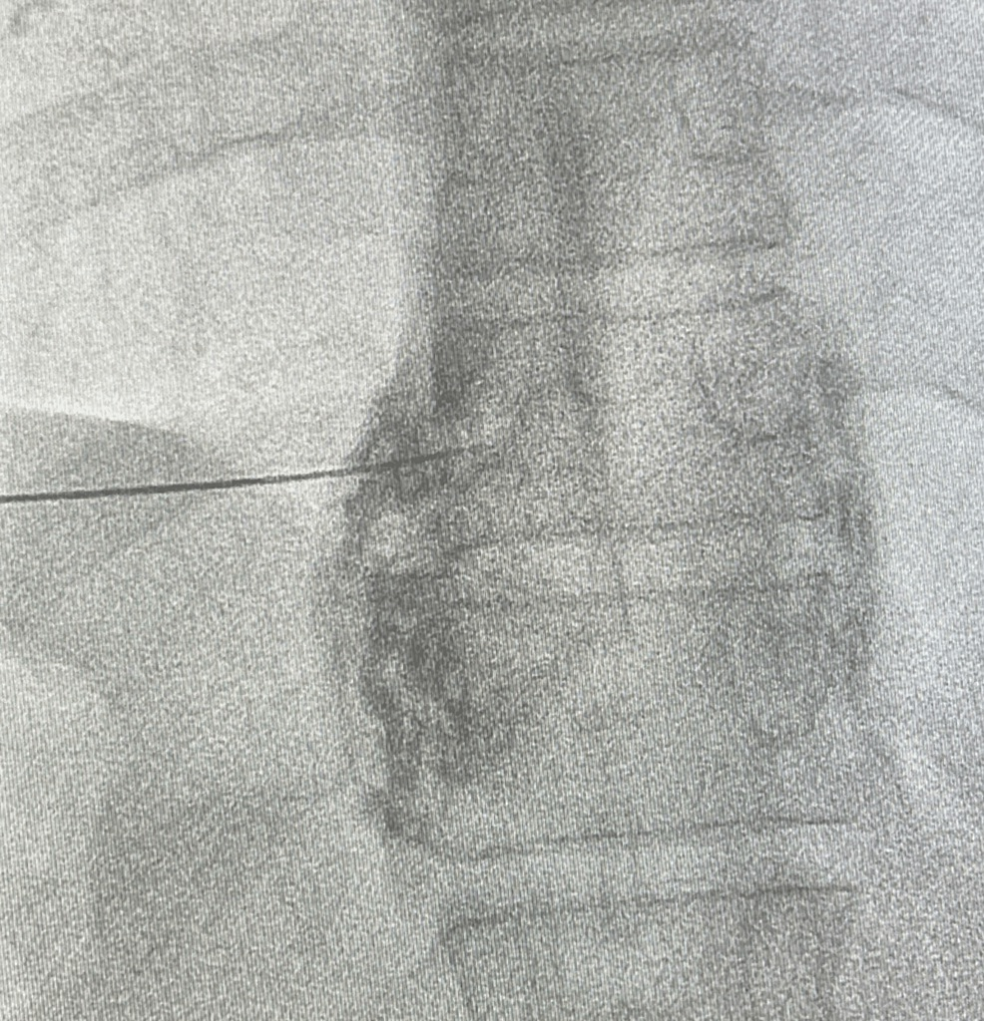
Injection of Non-Ionic Contrast in PA View to Confirm Needle Placement

The needle is then advanced to the junction of the anterior one-third and posterior two-thirds of the vertebral body in the lateral view (Figure 2). A curved needle if used is advanced so that the curve faces the medial side. After needle placement, the position is confirmed by injecting non-ionic contrast to rule out intravascular or intrapleural placement (Figure 3) [25].

Diagnostic blocks

Diagnostic blocks are administered prior to neurolysis and radio-frequency ablations. These are done to reduce injury to nerves and to confirm that pain is mediated through a visceral pathway [26]. Once the needle is appropriately placed, 10-15 ml of lidocaine (0.5-2%) or 10-15 ml of bupivacaine (0.125-0.25%) are injected in divided doses after negative aspiration to rule out intravascular injections. Post adequate pain relief following diagnostic blocks, neurolysis or radio-frequency ablation are carried out [14].

Neurolysis

Phenol and alcohol are the neurolytic agents used mainly for splanchnic nerve blocks. Alcohol concentrations used varies from a range of 50-100%. It acts by fatty substance extraction, denaturation of proteins, and causing precipitation of lipoproteins and mucoproteins. This causes Wallerian degeneration of Schwann cells and nerve cells. Instillation of alcohol is preceded by injection of local anesthetics to decrease the severe, transient pain caused by alcohol instillation. It causes more severe nerve destruction than phenol [27].

Phenol is viscous and painless on injection, has immediate local effect, is slower in onset, has shorter duration, and is available in concentrations of 6-10%. On administration, phenol in water causes coagulation of proteins and neural structure necrosis [28].

The volume of injectate is usually 25 ml on each side, but can range between 16-80 ml. The volume is dependent on the positioning of the needle and the ease of flow in the area. Smaller injectates are required when proximity of the needle and the nerves are very close [29]. 

Radio-frequency ablation

Radio-frequency ablation is a neuro-destructive procedure that uses temperature above 45-degrees Celsius to lesion the nerve plexus [30]. There is only 1 mm spread of lesion beyond the uninsulated tip with a cross-sectional lesion diameter of 5-6 mm . This minimizes the possibility of damage to nerve roots, epidural, and subarachnoid structures. Sensory and motor stimulation are performed to make sure no breakage in insulation. The radio-frequency needle is placed in the position as for diagnostic blocks and the stylet is removed. The radio-frequency probe is then advanced through the needle. Impedance is maintained at less than 25 ohms [31,32]. After sensory and motor stimulation, 2 ml of lidocaine 2% is injected before ablation. Lesioning is done for 60 seconds at 85 degrees and is repeated after rotating the needle 180 degrees. 2-5 ml of bupivacaine 0.5% is administered for post-procedure analgesia [25,33,34].

Complications

The complications associated with splanchnic nerve block are rare and include hypotension, pneumothorax, chylothorax, abdominal symptoms, acute alcohol intoxication symptoms, infection, paraesthesia and nerve damage, vascular injection and damage, epidural or subarachnoid injections, organ puncture, and drug allergies [35,36]. As this as a sympathetic block, potential for hypotension is pretty high and fluid prophylaxis is necessary. Pneumothorax is the most feared complication and the patient should be counseled to present to the emergency department in the event of developing shortness of breath [22].

## Conclusions

Abdominal pain in patients with abdominal cancer are debilitating in nature and associated with increased morbidity. Pharmacological therapies for alleviating this pain are often rendered inadequate and patients require increasing does of narcotics. Radio-frequency ablation and chemical neurolysis of the celiac plexus and splanchnic nerves have been proven to be beneficial. Radio-frequency ablation uses high frequency alternating currents at high temperatures to bring about coagulation of the nerves. There is denaturation of the cellular proteins due to high temperature, causing nerve destruction. Alcohol or phenol neurolysis result in sympathetic denervation by inflammation and also causes necrosis of the nerves. Both methods have been shown to be highly effective in relieving pain, reducing narcotic use, and improving patients' daily activity and quality of life.

Celiac plexus and splanchnic nerve blocks are good targets for neurolysis or radio-frequency ablation in abdominal cancers. The splanchnic nerve block is used in cases where celiac plexus blocks are difficult to perform owing to distorted anatomy due to tumors or associated lymphadenopathy. Given the nature of the debilitating pain associated with these conditions and inadequate pain relief with narcotics, these blocks are a boon in disguise to such patients with altered anatomy. Post administration of the splanchnic block, the functioning and quality of life of patients with abdominal malignancies improve. Hence, these blocks can be used to decrease the morbidity associated with abdominal malignancies. 

## References

[1] Swarm RA, Abernethy AP, Anghelescu DL (2013). Adult cancer pain. J Natl Compr Canc Netw.

[2] Cohen MZ, Easley MK, Ellis C (2003). Cancer pain management and the JCAHO's pain standards: an institutional challenge. J Pain and Symptom Manage.

[3] Goudas LC, Bloch R, Gialeli-Goudas M, Lau J, Carr DB (2005). The epidemiology of cancer pain. Cancer Invest.

[4] Svendsen KB, Andersen S, Arnason S (2005). Breakthrough pain in malignant and non-malignant diseases: a review of prevalence, characteristics and mechanisms. Eur J Pain.

[5] Temel JS, Greer JA, Muzikansky A (2010). Early palliative care for patients with metastatic non-small-cell lung cancer. N Engl J Med.

[6] Shaikh S (2019). The practice of cancer pain: a case series. Essentials of Interventional Cancer Pain Management.

[7] Stjernswärd J, Colleau SM, Ventafridda V (1996). The World Health Organization cancer pain and palliative care program: past, present, and future. J Pain Symptom Manage.

[8] Rahman A, Rahman R, Macrinici G, Li S (2018). Low volume neurolytic retrocrural celiac plexus block for visceral cancer pain: retrospective review of 507 patients with severe malignancy related pain due to primary abdominal canceror metastatic disease. Pain Physician.

[9] Vissers KC, Besse K, Wagemans M (2011). 23. Pain in patients with cancer. Pain Pract.

[10] Christo PJ, Mazloomdoost D (2008). Interventional pain treatments for cancer pain. Ann N Y Acad Sci.

[11] Al-Jumah R, Urits I, Viswanath O, Kaye AD, Hasoon J (2020). Radiofrequency ablation and alcohol neurolysis of the splanchnic nerves for a patient with abdominal pain from pancreatic cancer. Cureus.

[12] Dong D, Zhao M, Zhang J (2021). Neurolytic splanchnic nerve block and pain relief, survival, and quality of life in unresectable pancreatic cancer: a randomized controlled trial. Anesthesiology.

[13] Kambadakone A, Thabet A, Gervais DA, Mueller PR, Arellano RS (2011). CT-guided celiac plexus neurolysis: a review of anatomy, indications, technique, and tips for successful treatment. Radiographics.

[14] Singh-Radcliff N (2012). Celiac plexus blocks and splanchnic nerve blocks. Interventional Pain Medicine.

[15] Ahmed A, Arora D (2017). Fluoroscopy-guided neurolytic splanchnic nerve block for intractable pain from upper abdominal malignancies in patients with distorted celiac axis anatomy: an effective alternative to celiac plexus neurolysis - a retrospective study. Indian J Palliat Care.

[16] Garry RC (1957). Innervation of abdominal viscera. Br Med Bull.

[17] Yang HJ, Gil YC, Lee WJ, Kim TJ, Lee HY (2008). Anatomy of thoracic splanchnic nerves for surgical resection. Clin Anat.

[18] Candal R, Reddy V, Samra NS (2022). Anatomy, Abdomen and Pelvis, Celiac Ganglia. https://www.ncbi.nlm.nih.gov/books/NBK538129/.

[19] McCausland C, Sajjad H (2022). Anatomy, Back, Splanchnic Nerve. Anatomy, Back, Splanchnic Nerve. :6.

[20] Loukas M, Klaassen Z, Merbs W, Tubbs RS, Gielecki J, Zurada A (2010). A review of the thoracic splanchnic nerves and celiac ganglia. Clin Anat.

[21] Biologydictionary.net Editors (2022). Sympathetic nervous system. Biology Dictionary. Biologydictionary.net, February 23.

[22] Petersohn JD (2011). 42 - Sympathetic neural blockade. Pain Procedures in Clinical Practice, 3rd ed.

[23] Agbenyefia P, Stuart R, Chen G (2022). Sympathetic blocks: celiac plexus nerve block and neurolysis. Anesthesiology In-Training Exam Review.

[24] Shwita AH, Amr YM, Okab MI (2015). Comparative study of the effects of the retrocrural celiac plexus block versus splanchnic nerve block, C-arm guided, for upper gastrointestinal tract tumors on pain relief and the quality of life at a six-month follow up. Korean J Pain.

[25] Trescot A (2015). Splanchnic nerve blocks. Atlas of Pain Medicine Procedures.

[26] Vorenkamp KE, Dahle NA (2011). Diagnostic celiac plexus block and outcome with neurolysis. Tech Reg Anesth Pain Manag.

[27] Koyyalagunta D, Engle MP, Yu J, Feng L, Novy DM (2016). The effectiveness of alcohol versus phenol based splanchnic nerve neurolysis for the treatment of intra-abdominal cancer pain. Pain Physician.

[28] Racz GB, Holubec JT (1989). Stellate ganglion phenol neurolysis. Techniques of Neurolysis.

[29] Raj PP (2016). Block and lesioning of the splanchnic nerves. Techniques of Neurolysis.

[30] Tatli S, Tapan U, Morrison PR, Silverman SG (2012). Radiofrequency ablation: technique and clinical applications. Diagn Interv Radiol.

[31] Kapural L (2015). Radiofrequency ablation of splanchnic nerves for control of chronic abdominal pain. Tech Reg Anesth Pain Manag.

[32] Thapa D, Ahuja V, Gombar S, Ramakumar N, Dass C (2017). Radiofrequency ablation of bilateral splanchnic nerve in acute pancreatitis pain: interventional approach. J Anaesthesiol Clin Pharmacol.

[33] Zaky S, Abd-Elsayed A (2017). Splanchnic nerve radiofrequency ablation for treating resistant abdominal pain. Saudi J Anaesth.

[34] Mansano AM (2020). Splanchnic block and radiofrequency ablation. Interventional Pain: A Step-by-Step Guide for the FIPP Exam.

[35] Subramaniam S, Rajendran A, Gobinath M, Raghulraj G, Bhaskar M (2021). Effects of the celiac plexus block versus splanchnic nerve block for upper abdominal tumors on pain relief and quality of life-randomized comparative study. Int J Sci Stud En ligne.

[36] Afonso GL, Cardoso MG de M, Coelho IP, Cardoso BG (2016). Acute alcohol intoxication: uncommon complication associated to celiac plexus neurolysis during open surgical procedure in patient with refractory cancer pain. Case report. Rev Dor.

